# Plant-based expression platforms to produce high-value metabolites and proteins

**DOI:** 10.3389/fpls.2022.1043478

**Published:** 2022-11-08

**Authors:** Aditya Kulshreshtha, Shweta Sharma, Carmen S. Padilla, Kranthi K. Mandadi

**Affiliations:** ^1^ Texas A&M AgriLife Research and Extension Center, Weslaco, TX, United States; ^2^ Department of Veterinary Pathology, Dr. GCN College of Veterinary & Animal Sciences, CSK Himachal Pradesh Agricultural University, Palampur, India; ^3^ Department of Plant Pathology and Microbiology, Texas A&M University, College Station, TX, United States; ^4^ Institute for Advancing Health Through Agriculture, Texas A&M AgriLife, College Station, TX, United States

**Keywords:** plant secondary metabolites, heterologous production, molecular farming, plant-made secondary metabolites, plant-made therapeutic proteins

## Abstract

Plant-based heterologous expression systems can be leveraged to produce high-value therapeutics, industrially important proteins, metabolites, and bioproducts. The production can be scaled up, free from pathogen contamination, and offer post-translational modifications to synthesize complex proteins. With advancements in molecular techniques, transgenics, CRISPR/Cas9 system, plant cell, tissue, and organ culture, significant progress has been made to increase the expression of recombinant proteins and important metabolites in plants. Methods are also available to stabilize RNA transcripts, optimize protein translation, engineer proteins for their stability, and target proteins to subcellular locations best suited for their accumulation. This mini-review focuses on recent advancements to enhance the production of high-value metabolites and proteins necessary for therapeutic applications using plants as bio-factories.

## 1 Introduction

Many plant-based expression systems were developed for the large-scale production of valuable proteins and metabolites used in the pharmaceuticals, nutraceuticals, and cosmetics industries. These platforms are relatively cost-efficient, free from pathogens affecting humans, can synthesize complex proteins with post-translational modifications, and are scalable (Buyel, 2019; Burnett and Burnett, 2020). They can be used to produce vaccines, antibodies, antimicrobial peptides, hormones, growth factors, and industrially essential enzymes. Plants can be also used to produce high-value secondary metabolites (SMs). The SMs are produced from primary metabolic pathways and are induced in limited quantities during plant growth, development, and abiotic/biotic interactions (Chen et al., 2022). SMs have broad activities against viral, bacterial, and fungal infections and are used to treat various diseases like cancer, arthritis, diabetes, and neurological and respiratory disorders (De Filippis, 2016). Since chemical synthesis is expensive and challenging for many SMs, pharmaceutical industries depend on medicinal plants for sourcing SMs (Isah, 2019). With advancements in molecular techniques and synthetic biology tools, researchers have increased the quantity and quality of plant-made recombinant proteins and SMs. This mini-review summarizes different strategies for the enhanced production of valuable proteins and metabolites using plants as bio-factories for heterologous expression.

## 2 Heterologous expression of recombinant proteins in plants

Plant molecular farming is the practice of using plant-based platforms to produce high-value recombinant peptides and proteins. These proteins can either be stably or transiently produced, and as per need, they can be directed to accumulate in whole plants, seeds, chloroplasts, fruits, or roots (Xu et al., 2018). Moreover, many plant transformation methods based on *Agrobacterium* or polyethylene glycol (PEG)-mediated transformation, particle-bombardment, vacuum, and virus-based infiltrations are well established (Joung et al., 2015).

### 2.1 Approaches to enhance expression of recombinant proteins

#### 2.1.1 Promotor engineering and combinatorial stacking

The promoter is an essential element in regulating transgene expression. As per the need, many different types of promoters, like constitutive, inducible, tissue-specific, and synthetic promoters, are employed. The cauliflower mosaic virus 35S promoter (CaMV 35S), a strong constitutive promoter in either single or multiple copies, is widely used in dicot plants, whereas the maize ubiquitin-1 (Ubi-1) promoter is used to express therapeutic proteins in monocots (Phakham et al., 2021; Mirzaee et al., 2022). Recently, Damaj et al. (2020) reported a combinatorial stacked promoter system to enhance the expression of recombinant bovine lysozyme (BvLz). BvLz is a potent broad-spectrum antimicrobial enzyme used in the food, cosmetic, and agricultural industries. Combinatorial plant transformation and co-expression of BvLz under the control of various constitutive and culm-regulated promoters yielded high levels of expression (up to 11.5% of total soluble protein) in sugarcane culms (Damaj et al., 2020). Similarly, Padilla et al. (2020) successfully enhanced the expression of recombinant *Galanthus nivalis* L. (snowdrop) agglutinin (GNA) in sugarcane and energy cane. GNA possesses antiviral, antifungal, and antitumor activities. Under a single constitutive Ubi-1 promoter, GNA accumulated 0.04% and 0.3% of total soluble protein (TSP) in sugarcane culms and leaves, respectively. Its expression was further increased to 1.8% TSP and 2.3% TSP in sugarcane and energy cane lines, respectively, by co-expressing recombinant GNA under multiple promoters (pUbi-1 and culm-regulated promoters from sugarcane dirigent5-1 and sugarcane bacilliform virus; pUBD5:GNA) from different expression vectors. Moreover, the expression of recombinant GNA in the triple promoter transgenic lines (pUBD5:GNA) was further boosted to 2.7% TSP by inducing promoter activity with salicylic acid (Padilla et al., 2020). These studies demonstrate the great potential of the inducible promoters and combinatorial promoter stacking system to increase the accumulation of high-value therapeutic proteins in plants.

#### 2.1.2 Codon optimization

The native amino-acid codon degeneracy enables the optimization of non-favorable codons within an open reading frame of a protein. Some mRNAs possess cryptic splicing sites, secondary structures, mRNA stability elements, and alternative translation start sites that may negatively affect protein translation and accumulation. The codon optimization process uses synonymous codons without altering the protein amino acid sequence to increase translational efficiency (Webster et al., 2017). For instance, through codon optimization, the expression of stem cell factor (SCF) for ex vivo RBC production increased 25- to 30-fold in tobacco Bright Yellow-2 (BY-2) cells (Wang et al., 2021). In another study, a codon-optimized BvLz was stably expressed in sugarcane culms (Damaj et al., 2020). Further, codon optimization and other approaches enhanced the expression of human interferon-gamma (IFNγ) *via* bamboo mosaic virus (BaMV) mediated transient expression in *Nicotiana benthamiana* (Jiang et al., 2019).

#### 2.1.3 Expression using plant virus vectors

Plant viruses infect many crops, ornamentals, and medicinal plant species (Kulshreshtha et al., 2017; Sharma et al., 2019). Many asymptomatic or inactivated viruses have been engineered as chimeric expression vectors to transiently express therapeutic proteins in different plant species. For instance, the tobacco mosaic virus (TMV) was employed to express the receptor binding domain (RBD) of the SARS-CoV-2 spike protein in glycoengineered *N. benthamiana.* After purification of the protein and vaccination, mice produced RBD-specific antibodies that neutralized the SARS-CoV-2 virus infection in Vero E6 cells (Maharjan et al., 2021). Likewise, the bean yellow dwarf virus (BeYDV) expressed SARS-CoV-2 RBD and basic fibroblast growth factor (bFGF) in two microalgae species; *Chlamydomonas reinhardtii* and *Chlorella vulgaris* (Malla et al., 2021). Similarly, the cowpea mosaic virus (CPMV) and the potato virus X (PVX) expressed vaccine candidates in the *N. benthamiana* against the hepatitis E virus and the influenza virus, respectively (Mardanova et al., 2017; Zahmanova et al., 2021). These findings underscore the utility of plant viruses to express a wide range of biologics to produce therapeutic molecules rapidly during pandemics.

#### 2.1.4 Suppression of RNA silencing

A potential limitation in any eukaryotic expression system is the inherent host RNA silencing mechanism that may suppress the expression of foreign genes. Fortunately, many plant virus-encoded suppressor proteins can be co-expressed with the gene of interest to suppress host silencing, increasing recombinant protein expression by several folds (Gao et al., 2013). For instance, the co-expression of tombusvirus P19 suppressor led to a 40% increase in the expression of truncated human IFNγ accumulation in *N. benthamiana* using a BaMV-based vector (Jiang et al., 2019). In another example, PVX and a P19 suppressor were used to express a fusion protein (lhmlt) of melittin peptide and gonadotropin-releasing hormone receptor (GnRHR) in *N. benthamiana.* The purified protein was functional and inhibited the cancerous cells in the MTT assay (Naseri et al., 2020). In a recent study, CRISPR/Cas9 was also employed to knockout *N. benthamiana* dicer-like proteins 2 and 4 (NbDCL2 and NbDCL4). The knockout plants produced 6.96 folds higher expression of human fibroblast growth factor 1 (FGF1) than wild-type plants (Matsuo, 2022).

#### 2.1.5 Optimization of downstream processing

To enable efficient purification of the recombinant proteins from plant cells, often downstream extraction and processing steps need to be optimized. In the extraction phase, tissue homogenization releases impurities like host cell proteins, enzymes, and phenolic compounds. The phenolic compounds form covalent complexes with recombinant protein in the presence of plant polyphenol oxidases (PPO) and could result in aggregation and precipitation of recombinant protein which ultimately reduces protein yield and quality. Furthermore, many plant proteases can degrade target proteins. These factors can be partially addressed by including broad-spectrum protease inhibitors and antioxidants in the extraction steps (Buyel et al., 2015). In addition, CRISPR/Cas9-based target editing of PPO genes in the host cells can be employed to enhance *in planta* expression of recombinant therapeutic proteins (González et al., 2020). Another alternative is to target proteins via. signal sequences to accumulate into specific subcellular compartments such as apoplast, chloroplast, and endoplasmic reticulum (Habibi et al., 2017). Recombinant proteins can subsequently be purified with affinity tags such as maltose-binding protein, glutathione S-transferase (GST), thioredoxin, staphylococcal protein A, and poly-histidine tag (Pina et al., 2021).

### 2.2 Approaches to optimizing post-translational modifications

Glycosylation is a key post-translational modification needed in many human therapeutic proteins. Glycosylation in plants differs from humans and possesses additional α 1,3 fucose and β 1,2 xylose modifications. This may affect the activity, stability, and immunogenic responses of the therapeutic protein (Grabowski et al., 2014). Therefore, transgenic plants and plant cell cultures were developed to remove plant-specific glycan and to introduce human glycosylation pathways to produce more complex proteins like monoclonal antibodies (Castilho and Steinkellner, 2012). For instance, antibodies (CAP256-VRC26) against human immunodeficiency virus type 1 were expressed in glycoengineered *N. benthamiana*. These antibodies showed equivalent neutralizing activity to mammalian-produced antibodies (Singh et al., 2020). Similarly, human-like glycosylated granulocyte colony-stimulating factor (G-CSF) was produced in *N. benthamiana* by co-expressing genes needed for human-specific O-glycosylated G-CSF (Ramírez-Alanis et al., 2018).

### 2.3 Examples of key plant-made therapeutic proteins

Many essential therapeutic proteins, vaccines, and monoclonal antibodies are produced in plant systems. A handful of these biologics are commercialized or in clinical trials (**Table 1**). The first plant-made drug approved for human use by U.S. Food and Drug Administration (FDA) is Elelyso (taliglucerase alfa). It was produced in genetically modified carrot cells by Protalix Biotherapeutics to treat heritable type I Gaucher’s disease (Fox, 2012). Another drug is ZMapp, a cocktail of three monoclonal antibodies produced in transgenic tobacco. Its administration completely cured Ebola infection in *Rhesush macaques* (Qiu et al., 2014). The other plant-made commercialized proteins include bovine trypsin (expressed in corn and marketed by Sigma Aldrich), human and animal growth factors (expressed in barley seeds and marketed by ORF Genetics), and recombinant human serum albumin (expressed in rice and marketed by ScienCell Research Laboratories). Recently, a large-scale phase 3 clinal trial of *N. benthamiana* produced a quadrivalent influenza vaccine that demonstrated substantial protection against influenza viruses in adults (Ward et al., 2020). These findings conclude that plant-made platforms to produce biopharmaceuticals have broad potential but need more research and optimizations to meet market demand.

**Table 1:** Examples of plant-made vaccines, antibodies, and enzymes at various human clinical stages.

Product	Plant	Development stage	Purpose	References
*Bacillus anthracis* vaccine	*N. benthamiana*	Phase 1	Protection from anthrax	Paolino et al., 2022
*Vibrio cholerae* vaccine	Rice	Phase 1	Protection from cholera	Yuki et al., 2022
COVID 19 vaccine	*N. benthamiana*	Phase 3	Protection from COVID19	Hager et al., 2022
TNF fusion protein (OPRX-106)	Tobacco BY2 cells	Phase 2a	Treatment of ulcerative colitis	Almon et al., 2021
Monoclonal antibodies against HIV and HSV	*N. benthamiana*	Phase 1	Protection from HIV-1, HSV-1, and HSV-2	Politch et al., 2021
Pegunigalsidase alfa (PEGylated, α-galactosidase A)	ProCellEx system	Phase 1/2	Treatment of Fabry disease	Schiffmann et al., 2019
ZMapp (a triple monoclonal antibody)	*N. benthamiana*	Phase 1	Treatment of Ebola virus disease	Mulangu et al., 2019
*Plasmodium falciparum* vaccine	*N. benthamiana*	Phase 1	Protection from malaria	Chichester et al., 2018
Taliglucerase alfa	Carrot cells	Phase 3	Treatment of Gaucher disease	Zimran et al., 2018
HIV-neutralizing antibody	*N. tabacum*	Phase 1	Protection from HIV	Ma et al., 2015
B-cell follicular lymphoma vaccine	*N. benthamiana*	Phase 1	treatment of Non-Hodgkin's lymphoma	Tusé et al., 2015
human acetylcholinesterase-R (PRX-105)	*N. tabacum* cell line	Phase 1	Treatment against Organophosphorous (OP) poisoning	Atsmon et al., 2015

## 3 Heterologous expression of secondary metabolites in plants

SMs are produced from primary metabolic pathways in response to growth, development, and biotic and abiotic stresses. Several SMs have utility as therapeutics for human diseases. Many plant expression platforms like cell/suspension culture, callus culture, organ culture, hairy root culture, shoot culture, and transgenics are established for the heterologous production of SMs (Fazili et al., 2022). However, the accumulation of SMs can be enhanced further by a better understanding and optimization of metabolic pathways, the rate-limiting step(s), and genetic regulations.

### 3.1 Approaches to enhance the production of secondary metabolites

#### 3.1.1 Overexpression of rate-limiting enzyme(s)

The metabolic pathways may have single or multiple rate-limiting steps, and the overexpression of crucial rate-limiting enzyme(s) may improve the production of the desired metabolite. For instance, *Atropa belladonna* ornithine decarboxylase (AbODC) is a rate-limiting enzyme in the biosynthesis of tropane alkaloids. Tropane alkaloids have several clinical uses such as treating Alzheimer’s disease, postoperative nausea, and motion sickness. Overexpression of AbODC significantly increased the accumulation of putrescine, N-methylputrescine, hyoscyamine, and anisodamine in *A. belladonna* hairy roots and transgenic plants (Zhao et al., 2020). Similarly, overexpression of hyoscyamine six hydroxylase, a key enzyme in the scopolamine biosynthetic pathway, increased the production of scopolamine alkaloid in the hairy roots of *Datura innoxia* (Li et al., 2020). Camptothecin is an FDA-approved pharmaceutically important monoterpenoid indole alkaloid having potent anticancer properties. The expression of two key camptothecin biosynthetic genes, OpG10H and OpSLS, greatly enhanced its production (up to 3.5 mg/g) in transgenic *Ophiorrhiza pumila* hairy roots (Shi et al., 2020).

#### 3.1.2 Overexpression of transcription factors

Transcription factors are critical regulators of many metabolic pathways, and their expression can enhance SMs production. The overexpression of OpWRKY2 activated a camptothecin pathway gene, OpTDC. It resulted in a three-fold increase in camptothecin accumulation in *Ophiorrhiza pumila* hairy roots (Hao et al., 2021). In another study, astragalosides production was increased in *Astragalus membranaceus* hairy roots by overexpressing Arabidopsis transcription factor MYB12, Production of anthocyanin pigment 1 (PAP1), and maize leaf color (Lc) transcription factors (Li et al., 2022).

#### 3.1.3 Overexpression of MicroRNAs

MicroRNAs (miRNAs) are small noncoding RNAs important for gene regulation. They play crucial roles in growth, development, and stress responses, as well as the regulation of secondary metabolite pathways (Hossain et al., 2022). Small RNA sequencing of three grape varieties with different anthocyanin and flavonoid content identified that the grape lines with high anthocyanin content abundantly express two miRNAs, miR828 and miR858. These miRNAs target the MYB114 transcription factor, which is a negative regulator of anthocyanin biosynthesis. Hence, overexpression of these miRNAs increased anthocyanin accumulation (Tirumalai et al., 2019). In another study, overexpression of miR156 isolated from *Medicago truncatula* increased 8.3 folds in total anthocyanins production in transgenic poplar plants compared to wild-type plants (Wang et al., 2020).

#### 3.1.4 Gene editing

CRISPR/Cas9 mediated targeted mutagenesis has been developed to enhance the production of valuable metabolites in several plant species. *Atropa belladonna* produces a small amount of hyoscyamine and its structural analogs, anisodamine, and scopolamine. Zeng et al. (2021) used CRISPR/Cas9 to disrupt hyoscyamine 6β-hydroxylase (AbH6H). The transgenic plants produced a significantly higher hyoscyamine content but without anisodamine and scopolamine alkaloids (Zeng et al., 2021). In another study, Karlson et al. (2022) used CRISPR/Cas9 to silence cinnamate-4-hydroxylase (C4H) for enhanced flavonoid production into *N. tabacum* cell suspension. C4H-silenced cells produced a significantly higher concentration of cinnamic acid, chlorogenic acid, pinostrobin, and naringenin than wild-type cells (Karlson et al., 2022).

#### 3.1.5 Expression in genetically engineered microalgae

Although not plants, microalgae which are also photosynthetic organisms can be leveraged to produce a broad range of secondary metabolites of pharmaceutical importance (Sreenikethanam et al., 2022). The *Cannabis sativa* plant naturally produces cannabinoids that treat nausea and vomiting caused by cancer chemotherapy, neuropathic pain, and spasticity. Furthermore, cannabinoids also possess anticancer properties (Mangal et al., 2021). Genetically engineered Microalgae were recently used for the heterologous production of cannabinoids (Bolaños-Martínez et al., 2022). Microalgae system is advantageous as they have limited growth needs such as CO_2_ and few organic compounds.

### 3.2 Examples of key plant-derived secondary metabolites

Plant SMs are useful as treatments for several diseases, including cancer, diabetes, COVID-19, arthritis, and neurological and cardiovascular disorders (De Filippis, 2016). To date, only paclitaxel has been produced on a commercial scale. It is included in the WHO list of essential medicines and is used to treat different cancers. Phyton Biotech is commercially producing paclitaxel (trade name Taxol^®^ by Bristol-Myers Squibb) using plant cell fermentation technology based on cell lines of *Taxus chinensis v. marei* (https://phytonbiotech.com/). The other SMs produced in different expression systems showed higher expression than native plants (**Table 2**), but more efforts are still needed to produce SMs at an industrial scale.

**Table 2:** Enhancement of secondary metabolites by various molecular approaches.

Molecular Approach	Gene/Transcription factor/miRNA	Plant	Culture type	Secondary metabolite	Fold increase	Reference
*Overexpression of key enzyme(s)*	Δ^24^-reductase	Fenugreek	Hairy roots	Diosgenin	3	Nasiri et al., 2022
	Columbamine O-methyltransferase	*N. tabacum*	Transgenic plant	Total alkaloids	1.09- 1.83	Tu TQ et al., 2022
	12-Oxophytodienoate reductase	*Echium plantagineum*	Hairy roots	Acetylshikonin	2	Fu et al., 2022
	Geranylgeranyl diphosphate synthase (CrGGPPS2)	*Catharanthus roseus*	Transgenic plant	Vindoline	1.2- 2.5	Kumar et al., 2020
				Catharanthine	1.3- 3.5	
				Vinblastine	1.25-1.5	
	Tobacco lipid transfer protein (NtLTP1)	Orange mint	Transgenic plant	Monoterpenes	-------	Hwang et al., 2020
	AtDXS, AtHDR, AtGGPS, JcCAS	*N. benthamiana*	Transgenic plant	Casbene	-------	Forestier et al., 2021
	Triterpene synthase, farnesyl diphosphate synthase, 1-deoxyxylulose 5-phosphate synthase	*Arabidopsis thaliana *	Transgenic plant	Squalene and botryococcene	2	Kempinski and Chappell, 2019
*Overexpression of transcription factors*	PgMyb308	*Punica granatum*	Hairy roots	Shikimate	4.8	Dhakarey et al., 2022
	VviNAC17	Grape berry	Cell suspension culture	Anthocyanin and flavonoids	2.5	Badim et al., 2022
	VaMyb40	*Vitis amurensis*	Callus culture	Stilbenes	3.4-4	Ananev et al., 2022
	VaMyb60				5.9-13.9	
	BcERF3	*Bupleurum chinense *	Hairy roots	Saikosaponins	------	Han et al., 2022
	SmMYB1	*Salvia miltiorrhiza*	Hairy roots	Total phenolic content	------	Zhou et al., 2021
	ZmLc	*Scutellaria baicalensis*	Hairy roots	Baicalin	3.24	Park et al., 2021
				Baicalein	3.42	
				Wogonin	3.46	
	AtPAP1			Baicalin	5.53	
				Baicalein	5.80	
				Wogonin	2.29	
*Overexpression of miRNAs*	miR156	Poplar	Transgenic plant	Anthocyanins	8.3	Wang et al., 2020
*Gene editing*	VvbZIP36	*Vitis vinifera*	Transgenic plant	Anthocyanins	--------	Tu M et al., 2022
	GmF3H1, GmF3H2 and GmFNSII-1	Soybean	Transgenic plant	Isoflavone	--------	Zhang et al., 2020

## 4 Conclusion

Despite the advantages of plant-based expression platforms, only a few commercial products passed the regulatory approvals and reached the market (Schillberg et al., 2019). In our perspective, a critical barrier to commercialization using plant-based expression platforms comes down to investment returns. Any profitable company wants products of high quality, reliability, and quantity at a low cost. Currently, the market favors mammalian and bacterial platforms as they have a long and successful history of making pharmaceuticals. Furthermore, these platforms have demonstrated batch-to-batch consistency and safety, which is especially important for drug formulations for human use. Plant-based expression platforms face higher capital costs for raw materials and infrastructure, downstream process optimization costs, lower market demand, public acceptance, more biosafety needs, and regulatory approvals. Industries often are wary of switching from well-established platforms to plant-based platforms. Plant-based expression platforms need to demonstrate greater net economic return compared to prokaryotic and mammalian systems to be competitive. With recent advancements in genetic and genomic tools, the heterologous production of high-value proteins and metabolites in plant expression systems has gained traction. The platforms can be deployed to mass-produce (scalable) biopharmaceuticals in a shorter timeframe and can be relatively cost-effective compared to other conventional cell culture-based systems. This is particularly helpful during rapid response situations such as during pandemics. New approaches have also allowed for improvements in target protein stability and accumulation, post-translational modifications, and downstream recovery/purification of the proteins. We anticipate increasing market demand for high-value therapeutics and bioproducts that can boost commercial interest in plant-based expression platforms.

## Author contributions

KM conceptualized and supervised the study. AK, SS, and CP conducted the study and prepared the manuscript. All authors contributed to the article and approved the submitted version.

## Funding

This study was supported in part by funds from USDA NIFA (HATCH 1023984), Texas A&M AgriLife Research Insect-vectored Disease Seed Grants (114185-96210), and the Texas A&M AgriLife Institute for Advancing Health Through Agriculture to KM.

## Conflict of interest

The authors declare that the research was conducted in the absence of any commercial or financial relationships that could be construed as a potential conflict of interest.

## Publisher’s note

All claims expressed in this article are solely those of the authors and do not necessarily represent those of their affiliated organizations, or those of the publisher, the editors and the reviewers. Any product that may be evaluated in this article, or claim that may be made by its manufacturer, is not guaranteed or endorsed by the publisher.
